# Mobile ECMO retrieval of patients during the COVID‐19 pandemic

**DOI:** 10.1111/aor.14030

**Published:** 2021-07-20

**Authors:** Eugen Widmeier, Daniel Duerschmied, Christoph Benk, Dawid Staudacher, Tobias Wengenmayer, Alexander Supady

**Affiliations:** ^1^ Department of Medicine III (Interdisciplinary Medical Intensive Care), Medical Center, Faculty of Medicine University of Freiburg Freiburg Germany; ^2^ Department of Cardiology and Angiology I, Heart Center University of Freiburg Freiburg Germany; ^3^ Department of Cardiovascular Surgery, Heart Center University of Freiburg Freiburg Germany; ^4^ Heidelberg Institute of Global Health University of Heidelberg Heidelberg Germany

**Keywords:** COVID‐19, ECMO, mobile ECMO retrieval

## Abstract

ECMO support is particularly resource‐intensive and should be provided in highly specialized centers. Occasionally, ECMO needs to be initiated in non‐ECMO centers by mobile ECMO retrieval teams. Subsequently, patients must be transferred on ECMO to the ECMO center. We report single‐center data from out‐of‐center initiations of ECMO during the COVID‐19 pandemic. From March 2020 through February 2021, nine patients were connected to ECMO before transfer to our center. Median travel distance (IQR) from the referring hospital to our center was 66 km (20‐92), median land travel time (IQR) was 51 minutes (26‐92). Personal protective equipment was available for all team members and used throughout the missions. No infections of team members with SARS‐CoV‐2 occurred. Three patients survived until hospital discharge. Median duration of ECMO (IQR) was 18 days (2‐78) in survivors and 19 days (9‐42) in non‐survivors, respectively. Out‐of‐center initiation of ECMO during the COVID‐19 pandemic was feasible and safe for patients and staff.

## Graphical abstract

The ECMO retrieval team travels by land or by air to the referring center, connects the patient to the ECMO circuit and returns to the ECMO center together with the patient by land or by air.

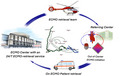

During the coronavirus disease 2019 (COVID‐19) pandemic, concerns have arisen that rationing of life‐saving therapies, such as mechanical ventilation or extracorporeal membrane oxygenation (ECMO), could be necessary due to a surge of patients overwhelming available resources and treatment capacities.^1^ ECMO support is particularly resource‐intensive and should therefore be provided in highly specialized centers, operating formally or informally within a so‐called “Hub and Spoke” concept.^2^, ^3^ Consequently, patient transfer capacity between hospitals according to the services needed must be provided to make the most effective use of available resources.^4^ Occasionally, patients requiring ECMO support must be transferred after out‐of‐center initiation of ECMO by a mobile ECMO retrieval team.^5^ While this approach has been successfully established in various ECMO centers before the outbreak of the COVID‐19 pandemic, data on the feasibility and the results of out‐of‐center initiation of ECMO during the pandemic are scarce.^6^

We report single‐center retrospective data of COVID‐19 patients supported with ECMO in our center after out‐of‐center initiation of ECMO and patient transfer by our ECMO retrieval team. We provide a 24/7 ECMO retrieval service. Our center is located in southwest Germany, bordering France and Switzerland (Figure 1). The geographic conditions result in our hospital serving as a major referral center for regional hospitals in a large area covering an approximately 100 km linear distance radius. Transport times between referring hospitals and our center occasionally exceed 2 hours when airborne transport is not possible.

**FIGURE 1:**
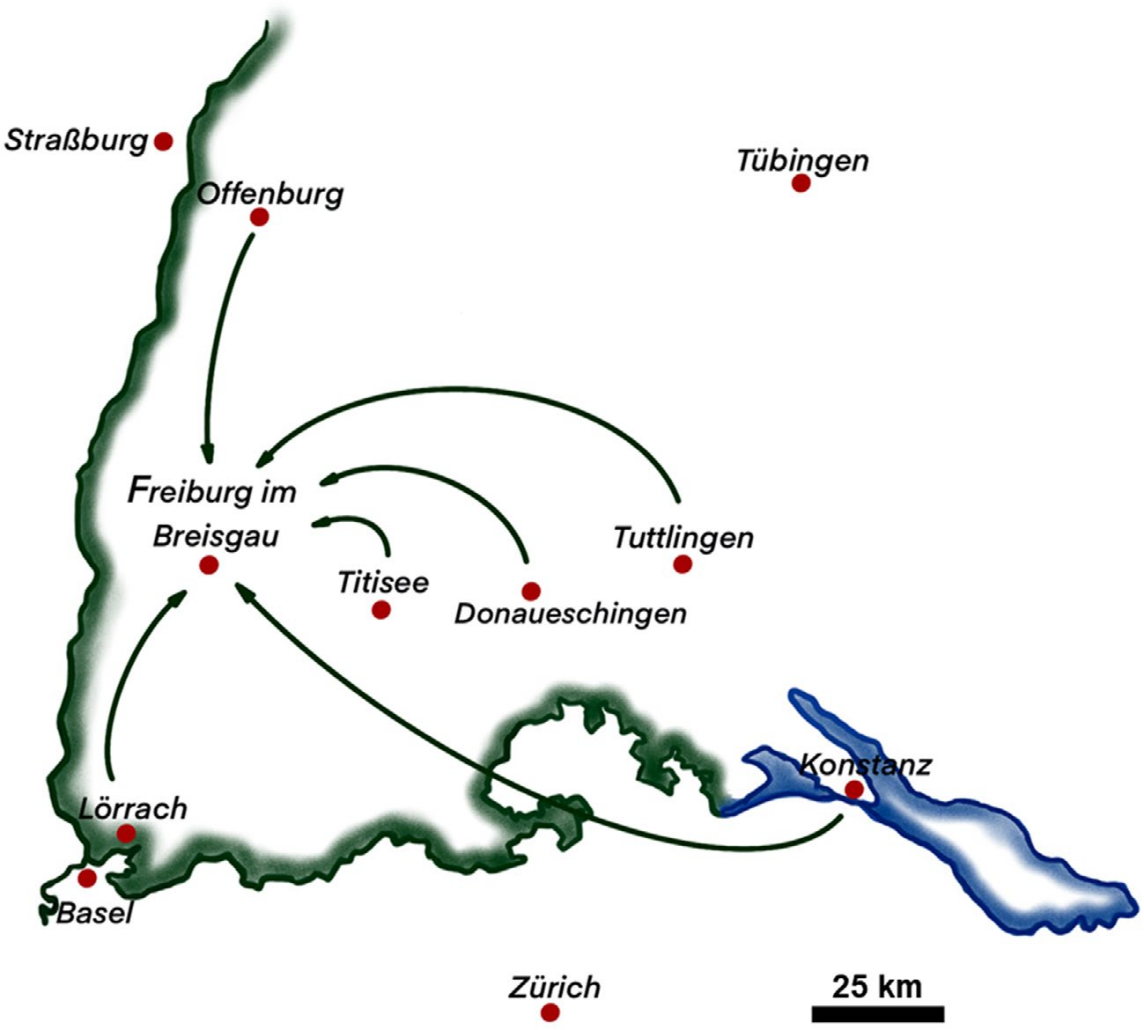
Sketched map of southwest Germany. Arrows connect referring centers with the University of Freiburg Medical Center. Between March 2020 and February 2021, 9 COVID‐19 patients received out‐of‐center extracorporeal membrane oxygenation (ECMO) in 7 different centers before transfer on ECMO to the University of Freiburg Medical Center [Color figure can be viewed at wileyonlinelibrary.com]

During the first 12 months of experience with treating COVID‐19 patients from March 2020 to the end of February 2021, 37 COVID‐19 patients were supported with veno‐venous ECMO in our center's medical intensive care unit (ICU). In 24% of these cases (9/37), ECMO support was initiated by our mobile ECMO retrieval team in the referring hospitals, and patients were transferred on ECMO (Table 1). The ECMO retrieval team reached out to the referring hospitals by land (6/9 [67%]) or by air (3/9 [33%]).

Median age (IQR) of the patients was 61 years (51‐65). Criteria for the indication of ECMO were based on previous recommendations (see Table 1 for blood–gas analyses and ventilator settings before initiation of ECMO).^7^ Sixty‐seven percent of the patients (6/9) were proned prior to ECMO, median duration (IQR) of invasive mechanical ventilation before ECMO was 2 days (1‐7). Vascular access was performed by an ultrasound‐guided percutaneous approach in Seldinger's technique, preferentially inserting a double‐lumen cannula into the right jugular vein.

Decision for out‐of‐center initiation of ECMO in contrast to patient transfer prior to cannulation to our center and in‐center initiation of ECMO was made by an experienced ECMO physician. When possible, timely transport without ECMO was sought, and only when this was deemed impossible the decision for out‐of‐center initiation of ECMO was made. During the COVID‐19 pandemic, we did not change our criteria for the initiation of ECMO. However, in times of particularly high numbers of patients with COVID‐19, we attempted to treat them in the referring hospitals for as long as possible to maintain sufficient capacity in our center for particularly severe cases. For some patients, this may have resulted in the need for out‐of‐center initiation of ECMO, which might have been prevented by a timely transfer as under prepandemic conditions.

All patients were transferred to our ECMO center by land. Median travel distance (IQR) from the referring hospitals to our center was 66 km (20‐92), median regular land travel time (IQR) was 51 minutes (26‐92). During the out‐of‐center missions and patient transfers to our center, the patients experienced no complications and no proven infection of medical personnel with the severe acute respiratory syndrome coronavirus 2 occurred. Personal protective equipment (FFP2 or FFP3 face masks, gowns, and protective goggles) was available and used throughout the mission by all team members and ambulance staff involved. Thirty‐three percent of the patients (3/9) survived until hospital discharge. Median duration (IQR) of ECMO was 18 days (2‐78) in survivors and 19 days (9‐42) in nonsurvivors, respectively. All deceased patients died on ECMO.

In our single‐center experience during the COVID‐19 pandemic, out‐of‐center initiation of ECMO was feasible, and patient transfer on ECMO provided by our mobile ECMO retrieval team was safe for all presented patients with COVID‐19 and staff. ECMO cannulation in the referring hospitals by the ECMO retrieval team was successful in all cases. No major complications occurred during cannulation or patient transfer to our center.

In extraordinary situations, such as during the COVID‐19 pandemic, the need for ECMO may increase significantly, and with it the need for out‐of‐center initiations and transfers of patients on ECMO.^1^, ^4^ Considering necessary protective measures for patients and staff, this approach is certainly possible but should ideally be performed by teams and within structures that have been previously established and routinely used under everyday conditions.

## 1 ETHICS APPROVAL AND CONSENT TO PARTICIPATE

Data collection was approved by the institutional ethics committee of the University of Freiburg (EK 151/14), the need for informed consent was waived.

## 2 COMPETING INTERESTS

AS and DD received speakers’ Honoraria from CytoSorbents, the manufacturer of the CytoSorb device. The Department of Cardiology and Angiology I received a research grant from CytoSorbents.

## AUTHOR CONTRIBUTIONS

All authors read and approved the final manuscript.

*Study design:* Supady

*Data collection:* Widmeier, Supady, Duerschmied, Benk, Staudacher, Wengenmayer

*Draft of the manuscript:* Widmeier, Supady

3

**TABLE 1:** Patient characteristics, treatment, and ventilation data before ECMO implantation

	Patient 1	Patient 2	Patient 3	Patient 4	Patient 5	Patient 6	Patient 7	Patient 8	Patient 9
Patient characteristics									
Age (years)	59	59	67	39	61	72	62	42	63
Sex	Male	Female	Male	Male	Female	Male	Female	Male	Female
BMI (kg/m^2^)	25.2	33.9	27.8	50.9	29.4	35.2	24.8	29.4	28.6
PMH	Burnout	Breast cancer (CR)	HTN	HTN	‐	HTN, DM, CHD	–	DM, infantile brain damage	CHD, bladder cancer
ICU survival	No	No	No	Yes	Yes	No	Yes	No	No
Days on ECMO	71	12	32	18	2	14	78	1	24
Vascular access	Jugular (double lumen cannula)	Jugular (double lumen cannula)	Jugular (double lumen cannula)	Femoro‐jugular	Bifemoral	Jugular (double lumen cannula)	Bifemoral	Jugular (double lumen cannula)	Jugular (double lumen cannula)
In‐hospital treatment before ECMO									
Duration of in‐hospital treatment before ECMO (days)	6	5	11	4	1	13	6	19	7
Duration of ICU‐treatment before ECMO (days)	6	5	8	4	1	10	6	17	5
Duration of invasive mechanical ventilation before ECMO (days)	2	5	1	3	1	8	0^a^	17	2
Prone positioning	Yes	Yes	No	Yes	No	Yes	No	Yes	Yes
Prognostic scores before ECMO									
SOFA	9	8	10	14	9	8	7	15	9
RESP	2	3	0	4	4	0	1	−4	−2
PRESERVE	3	1	7	1	6	2	4	4	5
Ventilator settings and arterial blood–gas analyses before ECMO									
Peak pressure (mbar)	30	30	32	32	40	26	–^a^	54	32
PEEP (mbar)	14	15	18	18	24	16	–^a^	16	14
Dynamic driving pressure (mbar)	16	15	14	14	16	10	–^a^	38	18
Tidal volume (mL)	450	550	480	500	450	390	–^a^	382	390
Breathing rate (L/min)	24	15	30	22	21	25	15	42	22
pH	7.3	7.4	7.1	7.2	7.4	7.2	7.5	7.1	7.2
PaO_2_ (mm Hg)	56.0	46.1	78.9	75.4	51.9	91.0	48.8	60.5	83.5
PaCO_2_ (mm Hg)	68.0	42,1	67.0	62.5	42.9	71.3	26.4	74.1	80.5
FiO_2_	1.0	1.0	0.9	0.9	1.0	1.0	0.85	1.0	1.0
PaO_2_/FiO_2_ (mm Hg)	56.0	46.1	87.7	83.8	51.9	91.0	57.4	60.5	83.5
Laboratory findings before ECMO									
Lactate dehydrogenase (U/L)	326	522	1129	680	535	747	740	1320	846
Aspartate aminotransferase (U/L)	79	45	76	278	31	96	36	702	62
Alanine aminotransferase (U/L)	36	36	39	138	28	56	22	506	27
Creatinine (mg/dL)	1.08	0.65	2.48	7.93	1.50	1.68	0.54	7.66	1.20
Troponin T (ng/L)	–	260	29	35	70	47	5	296	–
Troponin I (ng/L)	–	–	–	–	–	–	–	–	329
C‐reactive protein (mg/L)	316.8	482.3	502.8	362.4	130.9	394.6	195.2	112.0	61.4
Procalcitonin (ng/mL)	0.79	0.73	8.05	8.29	0.47	0.20	0.14	70.90	–
Interleukin‐6 (pg/mL)	–	540	3069	714	722	187	860	21	–
d‐dimers (mg/L FEU)	–	7.92	4.77	1.38	24.38	9.12	12.15	18.12	1.72
Vasopressor support before ECMO									
Norepinephrine (µg/kg/min)	0.286	0.222	0.741	0.518	0.118	0.037	0.000	0.556	0.346
Referring hospital									
Land travel distance to Freiburg University Medical Center (km)	66	66	92	92	35	127	4	4	73
Land travel time to Freiburg University Medical Center (minutes)	47	70	92	92	41	110	11	11	51
ECMO retrieval team transfer to referring hospital (by land or by air)	Land	Air	Air	Air	Land	Land	Land	Land	Land
Patient transfer to ECMO referral center (by land or by air)	Land	Land	Land	Land	Land	Land	Land	Land	Land

Abbreviations: BMI, body mass index; CHD, coronary heart disease; CR, clinical remission; DM, diabetes mellitus; ECMO, extracorporeal membrane oxygenation; FiO2, fraction of inspired oxygen; HTN, arterial hypertension; ICU, intensive care unit; PaCO2, partial pressure of arterial carbon dioxide; PaO2, partial pressure of arterial oxygen; PaO2/FiO2, ratio of the partial pressure of arterial oxygen to the fraction of inspired oxygen; PEEP, positive end‐expiratory pressure; PMH, past medical history; PRESERVE, Predicting Death for Severe ARDS on V‐V ECMO; RESP, Respiratory Extracorporeal Membrane Oxygenation Survival Prediction; SOFA, Sequential Organ‐Failure Assessment.

^a^ Patient was not on invasive mechanical ventilation before ECMO.

## Data Availability

All data will be available from the corresponding author on reasonable request.
